# Neuroinflammation represents a common theme amongst genetic and environmental risk factors for Alzheimer and Parkinson diseases

**DOI:** 10.1186/s12974-022-02584-x

**Published:** 2022-09-08

**Authors:** Rachel J. Boyd, Dimitri Avramopoulos, Lauren L. Jantzie, Andrew S. McCallion

**Affiliations:** 1 McKusick-Nathans Department of Genetic Medicine, Johns Hopkins University School of Medicine, Baltimore, MD 21205 USA; 2 Department of Pediatrics, Johns Hopkins University School of Medicine, Baltimore, MD 21205 USA; 3 Department of Neurology, Johns Hopkins University School of Medicine, Baltimore, MD 21205 USA; 4 Department of Neurology, Kennedy Krieger Institute, Baltimore, MD 21205 USA; 5 Department of Medicine, Johns Hopkins University School of Medicine, Baltimore, MD 21205 USA

**Keywords:** Multifactorial phenotypes, Common genetic disease, Neuroinflammation, Neurodegeneration, Alzheimer disease, Parkinson disease, Inflammatory response, Gene-by-environment interactions, Chronic inflammation

## Abstract

Multifactorial diseases are characterized by inter-individual variation in etiology, age of onset, and penetrance. These diseases tend to be relatively common and arise from the combined action of genetic and environmental factors; however, parsing the convoluted mechanisms underlying these gene-by-environment interactions presents a significant challenge to their study and management. For neurodegenerative disorders, resolving this challenge is imperative, given the enormous health and societal burdens they impose. The mechanisms by which genetic and environmental effects may act in concert to destabilize homeostasis and elevate risk has become a major research focus in the study of common disease. Emphasis is further being placed on determining the extent to which a unifying biological principle may account for the progressively diminishing capacity of a system to buffer disease phenotypes, as risk for disease increases. Data emerging from studies of common, neurodegenerative diseases are providing insights to pragmatically connect mechanisms of genetic and environmental risk that previously seemed disparate. In this review, we discuss evidence positing inflammation as a unifying biological principle of homeostatic destabilization affecting the risk, onset, and progression of neurodegenerative diseases. Specifically, we discuss how genetic variation associated with Alzheimer disease and Parkinson disease may contribute to pro-inflammatory responses, how such underlying predisposition may be exacerbated by environmental insults, and how this common theme is being leveraged in the ongoing search for effective therapeutic interventions.

## Introduction/background

The foundational characteristic of a multifactorial phenotype is the integrated role of complex genetic and environmental contributions. Both modulate the capacity of a system to maintain homeostasis; therefore, it makes sense that genetic and environmental factors may mediate their effects through overlapping biological avenues. Unraveling this complexity drives the search for a biological premise to unify both genetic and environmental contributions to disease risk and progression [1]. The role played by the inflammatory response in common, neurological disease risk represents one such unifying premise. By highlighting both established and emerging data implicating genetic and environmental drivers of inflammation in multiple neurodegenerative disorders, we explore what these data suggest in the context of understanding disease risk and how they can inform the development of a new generation of therapeutic approaches and treatment strategies.

Neurological disease risk has been explored by many genetic and epidemiological studies. Common disorders, including Parkinson disease (PD) and Alzheimer disease (AD), display complex modes of inheritance with risk influenced by both genetic and environmental factors. In many instances, familial forms of these disorders have provided valuable insight into the corresponding role of genetics. However, with most patients presenting as isolated cases, and with a respective prevalence of PD and AD in 5% and 35% of Americans over 85 years of age [2, 3], there is a clear role for common variation contributing to disease risk. Genome wide association studies (GWAS) have been widely employed and have identified a wealth of loci contributing to these diseases. The most recent, well-powered studies of PD and AD have identified over 90 and 50 risk loci, respectively [4, 5]. However, as with all complex, common phenotypes, genetic background alone explains only a fraction of the phenotypic variance[6]. In these neurodegenerative disorders, heritability ranges from 0.23 to 0.79 [7, 8], where the remaining variance may be accounted for by environmental factors and gene-by-environment (GxE) interactions.

GxE interaction is a common feature of complex traits; defined as an interdependency between genotype and environmental effect on phenotypic outcome [9]. Although many studies are not designed to incorporate specific environmental contributions in a genotype-dependent manner, in this review, we will highlight where interactions have been established in the human population, revealed in model systems, or may be reasonably inferred. Neurodegenerative disorders are subject to the influence of genetic variation and the environment, such that no independent insult is fully predictive of phenotypic outcome. However, the effects of many genetic and environmental risk factors of AD and PD appear to coalesce upon the modulation of immune surveillance and inflammatory response.

The importance of a well-functioning immune system is underscored in the face of infection or physical trauma. The innate immune system is the first line of defense against environmental insult, where mucus membranes, epithelial cells, anti-microbial peptides, and acid secretions provide protective, physical, biological, and chemical barriers between human organ systems and the outside world. In the event of tissue damage or breach by a foreign antigen, immune response cascades trigger an acute inflammatory response aimed to clear intruders and promote healing. During the onset of a normal inflammatory response, innate immune cells, including tissue-specific macrophages, dendritic cells, mast cells, neutrophils, and other circulating lymphocytes, use surface receptors, such as toll-like receptors (TLRs), to recognize pathogen-associated molecular patterns (PAMPs) and damage-associated molecular patterns (DAMPs) [10]. In addition to PAMP/DAMP recognition, these sentinel cells can phagocytose pathogens and modulate the production of transcription factors [11]. This can lead to the production of pro-inflammatory cytokines that attract leukocytes, growth factors, and other immune modulators to the site of inflammation, as well as release anti-inflammatory cytokines that balance the pro-inflammatory response [11]. Mast cells also release other immune mediators, including histamine, which promotes vasodilation and vascular permeability [11]. This allows additional mediators of the inflammatory response to exit the blood stream and aid in pathogen elimination or tissue repair. While the classic symptoms of acute inflammation—heat, swelling, redness, and pain—demonstrate tissue repair and pathogen elimination in many organ systems, they do not typify acute or chronic inflammation in the central nervous system (CNS).

**Fig. 1:**
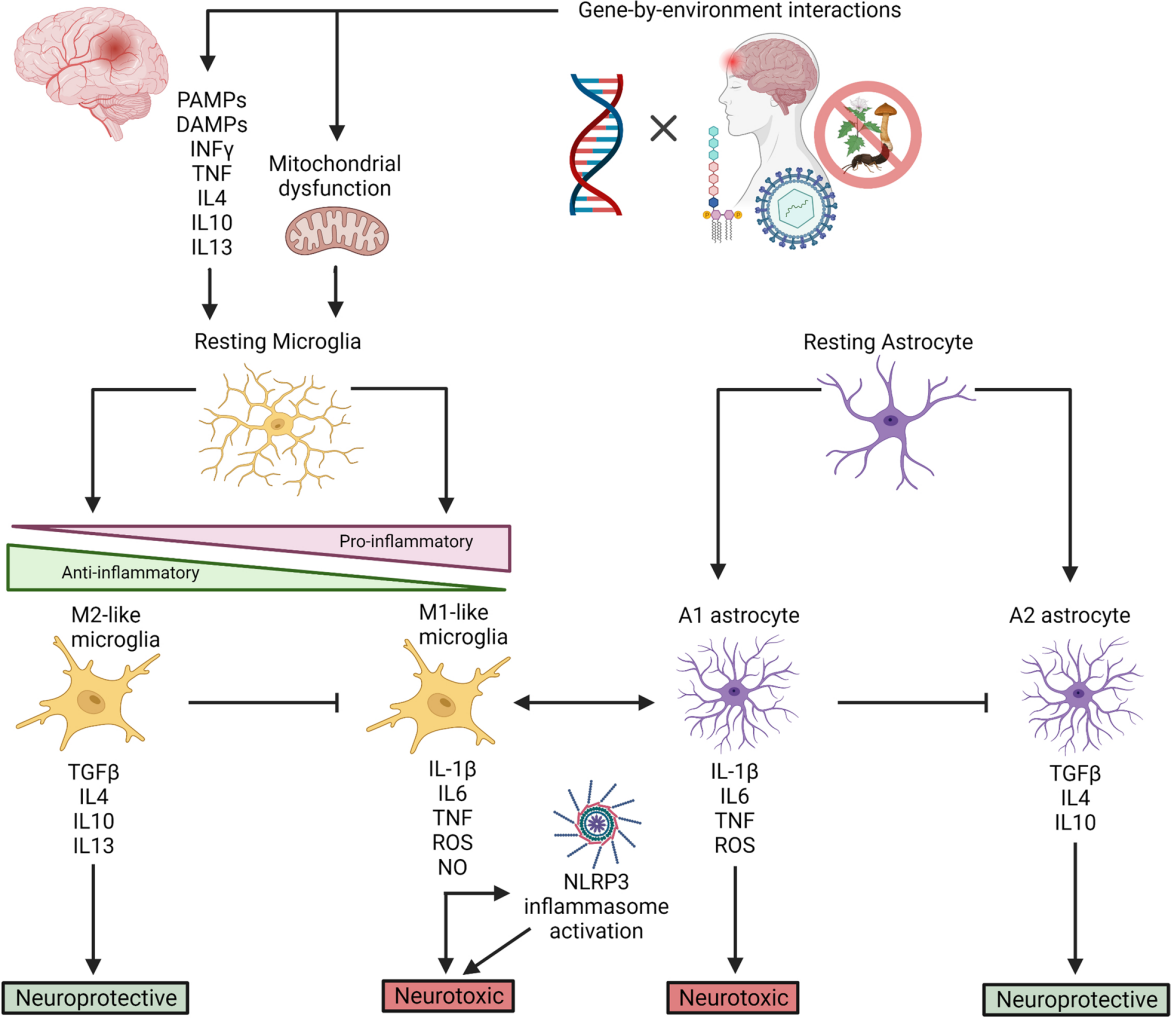
The neuroinflammatory response. Upon infection, insult, or injury to brain tissue and/or organelles, resting microglia are stimulated by PAMPs, DAMPs, reactive oxygen species (ROS), and cytokines. Activated microglia exist along a spectrum of pro-inflammatory (M1-like; neurotoxic) and anti-inflammatory (M2-like; neuroprotective) states, where pro-inflammatory cytokines (i.e., IL-1β, IL6, TNF), reactive oxygen species (ROS), free radicals (NO), and anti-inflammatory cytokines (i.e., TGFB, IL4, IL10, IL13) inform various neurotoxic and/or neuroprotective pathways. Pro-inflammatory cytokines, particularly IL-1β, can activate the NLRP3 inflammasome, which further modulates neurotoxic pathways. Cytokine release is also a means by which microglia communicate with one another and with astrocytes. Resting astrocytes are activated through such cellular communication with microglia, and exist as either A1 (pro-inflammatory) or A2 (anti-inflammatory) astrocytes, where cytokine release can cross-talk with microglia and further activate neurotoxic or neuroprotective pathways

Microglia are the resident, macrophagic immune cells of the CNS, with roles in surveillance and phagocytosis. TLRs on microglia recognize DAMPs and PAMPs, and these cells release inflammatory cytokines and second messengers which facilitate crosstalk with astrocytes and other immune cells (Fig. 1). Both microglia and astrocytes are major modulators of the innate immune response in the brain, and these cells can recruit other innate immune cells, such as neutrophils, dendritic cells, monocytes, infiltrating macrophages, and natural killer (NK) cells. Collectively, signaling from microglia and astrocytes can modulate cell-type specific transcriptional programs that can be neuroprotective or neurotoxic in nature (Fig. 1). Part of this innate branch of CNS immunity also involves the relationship between the complement cascade and glia, in which initiating components of the complement cascade mediate synapse removal by microglia in both developmental and disease states [12]. An immune response in the brain is also executed and regulated by adaptive immunity, in which subclasses of T-cells and B-cells can traffic to sites of inflammation, produce inflammatory cytokines, and modulate microglial processes.

The immune system, such as all natural cells, is developmentally regulated and changes throughout one’s lifespan. Therefore, the maintenance of this complex system, and its capacity to buffer insults, necessitates the contributions of many genes. Both inherited and somatic variation can impact the capacity to buffer stress over time; so too, the influence of environmental insult can acutely or chronically compound genetic destabilization through acquired mutation and cellular stress. These influences read out their effects differentially across -omic space, including the genome, transcriptome, proteome, metabolome, and epigenome [13]. Further, in the context of an individual’s genetic background, environmental exposures (so-called exposome) establish and modify the ensuing biological milieu, which lays the foundation for phenotypic variation [9]. To this end, both direct and indirect effects of genetic background and environmental challenge can modulate mission-critical immune genes or impose cellular stress with a net effect of elevating inflammatory propensity. These GxE effects work in concert to destabilize cellular homeostasis. When this delicate balance is sufficiently perturbed, the result can be detrimental.

Inappropriate signaling, hyperactive signaling, or failure to properly resolve an acute inflammatory response can elicit chronic inflammation that may result in a spectrum of disease phenotypes [14]. As such, there is increasing awareness that an inflammatory response can act as a sensitizing agent in the onset, exacerbation, and/or progression of genetic disease [15, 16]. The recognition of inflammation as a mode of broad homeostatic imbalance in the pathogenesis of neurodegenerative disorders may point toward more inclusive strategies in combatting disease for at-risk individuals. In this review, we discuss the progress made in understanding the role of the inflammatory response in the onset and progression of Alzheimer disease and Parkinson disease. While the data presented in this review do not preclude the contribution of non-inflammatory mechanisms to disease pathology, here, we draw together the growing body of evidence implicating immunomodulatory mechanisms as a unifying node of dysregulation in these neurodegenerative diseases. We discuss the role of both genetic and environmental mediators in disease-associated inflammatory dysfunction, as well as the prophylactic and therapeutic strategies that are being considered to restore this homeostatic imbalance. Collectively, these data may be used to define hypotheses with potential significance for understanding neurodegenerative disease risk and identifying novel therapeutic avenues.

## The genetic architecture of Alzheimer disease

Alzheimer disease (AD) is the most prevalent neurodegenerative disorder in the human population and underlies 60–70% of the 50 million dementia cases worldwide. Globally, AD is the seventh leading cause of death [17]. In the US alone, an estimated 6.2 million individuals (3.8 million women and 2.4 million men) are living with AD [3]. The global prevalence and incidence of AD is higher in women than in men [17], an observation that may be explained by the longer life expectancy of women than of men [18]. Clinically, AD is characterized by a progressive decline in cognition and productive behaviors—the ability to perform tasks, solve problems, and interact with others [19]. These debilitating symptoms of AD are mediated by hippocampal and cortical neuron loss, in addition to impairment and degeneration of the basal cholinergic system. While brain pathology is often characterized by progressive aggregation of extra-neuronal amyloid-beta (Aβ) plaques and intraneuronal neurofibrillary tangles (NFT) of hyperphosphorylated tau [20], there is a lack of consensus implicating protein aggregation as the central, pathogenic event in AD neurodegeneration [21].

AD can be classified as early onset AD (EOAD; age of onset ≤ 60), caused by rare genetic variants of strong effect, or late-onset AD (LOAD; age of onset ≥ 60), wherein risk is elevated by a spectrum of more common variants, of comparatively small effect [22]. EOAD only accounts for 2–10% of all AD cases, which are highly heritable (92–100%) within families [22, 23]. These familial cases are most often inherited in an autosomal dominant fashion that can be explained by mutations in the amyloid precursor protein (*APP*) and presenilin (*PSEN1/PSEN2*) genes; however, mutations in these three genes contribute to less than 1% of total AD cases [23]. LOAD is responsible for up to 98% of the remaining cases, which are sporadic/multifactorial, as genetic factors account for 53–79% of phenotypic variability [7].

Despite relatively high heritability in both forms of AD, only 30–33% of the phenotypic variance can be attributed to common single nucleotide polymorphisms (SNPs) [24]. The strongest known risk variant for both EOAD and LOAD is the ɛ4 allele of the gene encoding apolipoprotein E (*APOE*), although the presence of this variant is not sufficient to cause disease. Alone, three common *APOE* variants (ε2, ε3, and ε4) account for approximately 6% of the genetic contribution to AD risk [25], and the *APOE4* allele contributes 27.3% of the percent attributable fraction [26]. Compared to individuals who are homozygous for “wild-type” *APOE3*, individuals who are heterozygous or homozygous for *APOE4* are at threefold or eightfold increased risk for AD, respectively [27]. Those with an *APOE2* allele (ɛ2/ɛ2 or ɛ2/ɛ3 genotypes) have a protective effect against AD compared to other *APOE* genotypes (ɛ2/ɛ4, ɛ3/ɛ3, ɛ3/ɛ4, ɛ4/ɛ4) [27].

Aside from *APOE*, GWASs and corresponding meta-analyses have identified over 50 additional risk loci associated with AD risk [5, 28], at least 30 of which have been replicated in multiple studies [5]. Although the factors underlying AD risk are complicated by the sheer multiplicity and concomitant effects of these implicated loci, the observation that many AD risk loci are known or predicted to contribute to immunomodulation, has begun to emerge as a mechanistic theme. Moreover, a growing body of data now highlights the contributions of inflammation, as both a risk factor and pathological mechanism of AD, in this complex disorder.

### The role of inflammation in driving AD pathology

**Fig. 2:**
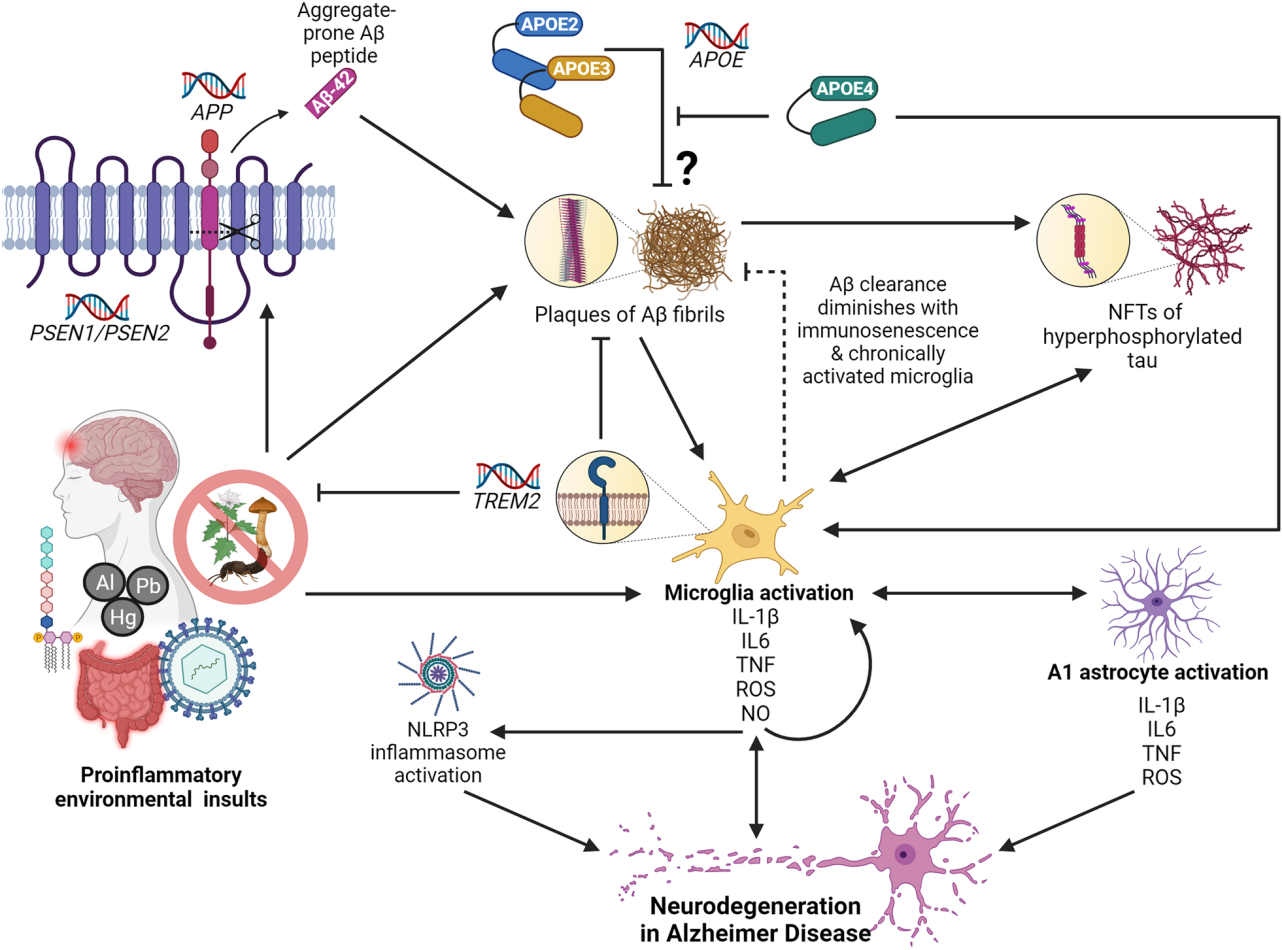
The role of neuroinflammation in Alzheimer disease (AD). Microglia activation, and the subsequent release of pro-inflammatory cytokines, ROS, and NO, is a central event in the onset of neurodegeneration in AD. Pro-inflammatory microglia release cytokines that cause a feed-forward loop of microglia activation, activate the NLRP3 inflammasome, and facilitate cross-talk with A1 astrocytes, all of which coalesce on neurodegenerative pathways. In AD, microglia can be activated upon detection of PAMPs, DAMPs, and other pro-inflammatory molecules as a result of environmental insult—including gut dysbiosis and sustained exposure to LPS endotoxin, viral infection, and TBI. These pro-inflammatory environmental insults are also associated with accelerated amyloid β (Aβ) plaque deposition and the overexpression of APP and β-secretase (*PSEN1/PSEN2*). Mutations in *PSEN1* and *PSEN2* can result in the cleavage of APP to produce 42-residue, aggregate-prone peptides, and mutations in *APP* can lead to overproduction, misfolding, and formation of Aβ fibrils. Microglia become activated upon sensing DAMPs from Aβ plaques, and are sequestered to clear them. However, chronically activated microglia and immunosenescence diminish the efficacy of this ability over time. TREM2 modulates microglia activity and survival. Mutations in *TREM2* can impact the ability of microglia to modulate cytokine production, phagocytose bacteria, and clear neural debris. When Aβ plaques are not cleared, they, and the chronically activated microglia, can induce the formation of NFTs. Hyperphosphorylated tau protein can further activate microglia. Although their exact mechanism of action is unknown, the APOE2 and APOE3 isoforms are protective against AD and are thought to play a role in clearing Aβ plaques. The APOE4 isoform, however, is unable to clear Aβ plaques and forms neurotoxic fragments that can activate microglia

Astrocytes and microglia are the resident immunomodulatory cell types in the brain (Fig. 1). Microglia are tissue-specific macrophages that, when activated, act as the first line of defense against pathogens and cellular damage. Microglia also function to stimulate myelin repair and remove neurotoxic protein aggregates [29]. As such, microglia become activated upon sensing DAMPs from Aβ plaques (Fig. 2). In the brain, microglial-specific receptors, including triggering receptor expressed on myeloid cells 2 (*TREM2*) and Sialic Acid-Binding Ig-Like Lectin 3 (*SIGLEC3/CD33*), modulate phagocytosis of Aβ by microglia [30, 31]. The activated microglia surrounding these plaques initiate proinflammatory cascades involving tumor necrosis factor (TNF) and interleukin 1β (IL-1β), that can trigger the NLR family pyrin domain containing 3 (NLRP3) inflammasome and lead to cell death and tissue damage [32]. In fact, studies of AD patients revealed increased numbers of activated microglia accompanied by elevated levels of TNF and IL-1β, and other pro-inflammatory cytokines [33]. Paradoxically, clearance of Aβ by microglia may exacerbate Aβ pathology: as chronically activated microglia release pro-inflammatory mediators and become even more pro-inflammatory, their capacity for clearing Aβ diminishes [32, 34] (Fig. 2). These observations are consistent with the knowledge that increased susceptibility to infection and disease result from age-related immune dysfunction (immunosenescence), and that age is the greatest risk factor for AD.

In the ageing brain, immunosenescence impedes the ability of microglia to resolve insult by reducing process length and arborized area of microglia [35]. This may diminish the phagocytic activities of microglia which, in turn, leads to a mounting density of Aβ plaques (Fig. 2). Thus, immunosenescence and Aβ plaque-associated microglia are among the main drivers of neuroinflammation in the progression of AD pathology. Aβ plaque deposition precedes the presence of NFTs in the AD brain. Microglial activation and the release of pro-inflammatory cytokines are sufficient to induce tau phosphorylation and lead to neurotoxicity through the production of reactive oxygen species (ROS) [36, 37] (Fig. 2). Elevated levels of ROS are inextricably linked to altered neuronal signaling, inflammatory response, and neuronal death. This is a vicious cycle, in which ROS and oxidative stress are both triggers and consequences of inflammatory response [38]. This inflammatory feedback loop can result in increasing cytokine production, which can signal to and recruit other immune cells, including T cells.

Upon stimulation, the production of microglial second messengers can also facilitate crosstalk with astrocytes (Fig. 1). “Protective” A2 astrocytes are characterized by the expression of neurotrophic factors and function to mediate tissue repair, defend against oxidative stress, and maintain neuronal homeostasis [39]. However, release of pro-inflammatory cytokines from activated microglia can lead to the activation of A1 astrocytes, characterized by an NFκβ-dependent program. This reinforces microglial activation, exacerbates neuroinflammation, and further contributes to an expanding cycle that includes oxidative stress, ROS production, neurotoxicity, and apoptosis [39] (Figs. 1, 2). Post-mortem studies of brain tissue from AD patients have revealed elevated levels of destructive A1 astrocytes, as well as upregulation of astrocytic γ-aminobutyric acid (GABA) [39, 40]. Animal models of AD have demonstrated that this excessive release of GABA from reactive astrocytes is associated with impairment of memory and synaptic plasticity [40]. Therefore, as the role of glia, immunosenescence, oxidative stress, and ROS are increasingly acknowledged in both initiating and contributing to AD pathology through inflammatory pathways, the mechanistic contributions of inflammation-pertinent genetic risk factors can be better evaluated.

### Genetic mediators of inflammatory response in AD

There is mounting evidence suggesting that genetically driven, immune-related processes are causative of AD pathology, but also elicit downstream effects of cell damage. One of the major functions of APOE, the strongest genetic predictor of AD risk, is to regulate lipid levels in plasma and tissues by serving as a ligand in low-density lipoprotein receptor-mediated endocytosis. In the CNS, APOE is predominantly expressed by astrocytes, but also by neurons and activated microglia, and is responsible for lipoprotein clearance and cholesterol transport to neurons. APOE is expressly involved in Aβ clearance; indeed, lipidated APOE binds soluble Aβ and facilitates Aβ uptake through cell-surface receptors [41]. However, the APOE4 isoform impairs Aβ clearance, relative to APOE3 and APOE2, which promotes a pro-inflammatory state (Fig. 2).

While murine ApoE lacks natural isotypes akin to human APOE2 and APOE4, transgenic (Tg) models expressing humanized APOE4 exhibit a significant increase in serum and brain levels of TNF and interleukin 6 (IL-6) [42], as well as elevated microglia nitric oxide (NO) release [43], compared to APOE3-Tg mice. These results have been recapitulated in human populations, showing that TNF, IL-6, and IL-1β levels in plasma are associated with AD patients possessing the ε4 allele [44], and that immune-activated, human monocyte-derived macrophages from APOE4 patients demonstrate a significant increase in NO production [43]. APOE3 plays an essential anti-inflammatory role in the CNS, while APOE4 amplifies the proinflammatory response induced by Aβ, thus resulting in a robust inflammatory phenotype that causes neuronal dysfunction. The mechanisms behind the immunomodulatory functions of APOE are not well-understood; however, it is theorized that higher retention of cholesterol in lipid rafts enhances TLR signaling in macrophages (i.e., microglia) [45]. APOE4 is reported to be less effective than APOE3 at inducing macrophage ATP binding cassette A1 (ABCA1) expression; thus, reducing cholesterol efflux and promoting cholesterol accumulation within the cell membrane of macrophages [46]. This may be due to the abnormal structure of the APOE4 isoform, caused by a single amino acid substitution (C112R) that results in a C–N domain interaction and decreases phospholipid binding capacity [47].

The amyloid precursor protein (APP) is cleaved by secretases and processed into the small Aβ peptides that embody pathognomonic protein aggregation in AD (Fig. 2). There are approximately 49 known mutations implicated in AD at the *APP* locus (https://www.alzforum.org/mutations/app). These mutations have recognized roles in Aβ overproduction and misfolding [48–50], which promotes plaque formation and onset of a robust, pro-inflammatory response, as described above. The pathological effects of *APP* overproduction are consistent with the high prevalence of AD and associated dementias in people with Trisomy 21, as *APP* is encoded on chromosome 21 [51]. Mutations in the presenilin genes (> 300 in *PSEN1*; > 55 in *PSEN2)* are also commonly implicated in familial AD (https://www.alzforum.org/mutations/psen-1; https://www.alzforum.org/mutations/psen-2). *PSEN1* and *PSEN2* encode subunits of γ-secretase, the enzyme involved in APP cleavage, and mutations in these genes most often result in an altered ratio between the aggregation-prone 42-residue Aβ peptide and the 40-residue variant (Fig. 2). The accumulating proportion of this aggregate-prone protein is sufficient to induce an inflammatory response that can be compounded by the effects of immunosenescence, oxidative stress, and ROS production. This ratio, together with APOE genotype, is used as an effective biomarker of AD progression [52].

The gene encoding *TREM2* is also consistently implicated in AD, and variants at this locus act as a modifier in AD risk [53]. TREM2 is a transmembrane, immunoglobulin receptor that modulates microglial activity and survival (Fig. 2). TREM2 is required for sensing DAMPs and plays key roles in the ability of microglia to phagocytose, clear neural debris, and produce anti-inflammatory cytokines [54]. In addition, TREM2 is a receptor for Aβ; thus, variants at this locus can contribute to AD risk through dysregulated microglial activation, Aβ clearance, and apoptosis [55]*.* In fact, *TREM2* haplodeficient mice exhibit larger, more diffuse, and less compact plaques—morphology that is shared by AD patients with *TREM2* loss-of-function variants [56].

Further to these examples, GWASs have uncovered a strong overlap between AD risk loci and a swath of immunomodulatory genes. While not as well-studied as *APOE*, many of these genes, including CD33, ATP Binding Cassette Subfamily A Member 7 (*ABCA7*), Clusterin (*CLU*), Complement receptor 1 (*CR1*), and CD2-Associated Protein (*CD2AP*), play immunomodulatory roles in AD. *ABCA7*, such as *ABCA1*, encodes a transmembrane lipid transporter primarily expressed in the brain by microglia. ABCA7 is required for the transport of proteins bound to APOE, as well as microglial clearance of Aβ [57]. The proteins encoded by *CD33* and *CLU* have been shown to mediate Aβ clearance by microglia [30, 31]. *CR1*, and other risk loci that are essential components of the complement cascade, similarly play roles recruiting microglia to clear Aβ, as well as modulating inflammation [58]. CD2AP regulates interactions between T-lymphocytes and antigen-presenting cells, and may modulate neuroinflammation in the context of chronic infection [59]. Additional AD risk loci, specifically the Human leukocyte antigen (HLA) locus and Inositol Polyphosphate Multikinase (*IPMK*), have been found to harbor variants that are pleiotropically associated with other immune-related diseases, such as Crohn disease and psoriasis [60]. Taken together, these findings suggest that AD risk can be mediated both by genetically driven inducers of inflammation, as well as by genetic variation resulting in dysfunction of immune-response pathways.

### Environmental mediators of inflammatory response in AD

The multifactorial nature of AD is highlighted by sex bias in disease incidence, variable age of onset, and variable expressivity of clinical presentation. Hence, in addition to the substantial contribution of genetic factors toward AD risk, disease onset and progression are further modulated by environmental factors. Many early cellular and molecular studies have emphasized the “amyloid cascade hypothesis,” suggesting that Aβ deposition marks the pathogenesis of AD [61]. In support of this, certain elements in the human exposome, termed “Alzheimerogens,” are thought to promote the production of Aβ plaques; thus, inciting a neuroinflammatory response that results in neurodegeneration [62]. For example, pesticides and herbicides have been recognized by epidemiological studies and meta analyses as environmental risk factors for AD/dementia [63, 64]. Rodents exposed to various pesticides showed signs of Aβ aggregation, tau hyperphosphorylation, neuroinflammation, neurodegeneration, and cognitive deficit [65, 66] (Fig. 2).

While Aβ accumulation remains both a potential trigger and consequence of neuroinflammation, AD is becoming increasingly recognized as a neurological disease in which immunosenescence and inflammation are major drivers and sensitizing factors of disease onset/progression [67]. Consistent with our underlying thesis, common environmental risk factors of AD and associated dementias can trigger an inflammatory response in the CNS that can tip the balance toward initiating disease and exacerbating the progression of AD neuropathology.

Traumatic brain injury (TBI) is a common neuroinflammatory insult experienced by over 69 million people worldwide—including military personnel, amateur and professional athletes, adults and children subject to falls, and individuals who have experienced motor vehicle collisions or other traumas/penetrating insults [68]. Compared to controls, individuals who have experienced moderate or severe TBI have a twofold or four-and-a-half-fold risk of developing AD, respectively [69]. Case–control and cohort studies have found that a history of head injury is associated with elevated risk of AD and dementia (OR = 1.58–2.29) [70, 71]. Critically, TBI can induce chronic neuroinflammation, as evidenced through the activation of microglia and the detection of pro-inflammatory biomarkers up to 12 months following a TBI [72–74] (Fig. 2). Furthermore, both human and rodent models demonstrate that there is a marked increase in the production of APOE and APP following a brain injury [75, 76], revealing a clear GxE interaction that may modulate AD risk (Fig. 2). In fact, individuals with the ε4 allele experienced worse recovery outcomes following TBI, compared to injured individuals lacking the ε4 allele [77]. Other studies suggest that aberrant APP processing can occur following a TBI, as evidenced by a greater density of Aβ plaques and NFTs in the postmortem brains of TBI patients, compared to non-injured controls [78]. In support of this, the deposition of Aβ plaques, and other neural changes, can be detected as early as 2 h following a TBI [79]. Interestingly, repetitive TBI can lead to chronic traumatic encephalopathy (CTE), a neurodegenerative condition that is also characterized by NFT and memory loss [80].

Chronic stress, including post-traumatic stress disorder (PTSD), is another common risk factor for AD. Furthermore, inflammatory response associated with a TBI may actually contribute to PTSD symptoms [81], suggesting a comorbidity, where inflammation is the common denominator [82]. Pro-inflammatory biomarkers are also seen in individuals who have experienced early life trauma and depression [83]. Notably, indicators of chronic stress, such as depression and hypertension, are considered risk factors for AD [84]. Furthermore, overexpression of corticotropin releasing factor, which modulates the stress response, revealed increased phosphorylation of β-secretase and tau in female mice [85]. These female mice exhibited increased Aβ plaque disposition and cognitive impairment relative to males, which mirrors female vulnerability to AD in human populations. The inflammatory response associated with chronic stress is not restricted to the brain, but also exists at the periphery, rendering AD at least partially systemic [67, 86].

The “endotoxin hypothesis of neurodegeneration,” also invokes an inflammatory foundation of AD, postulating that chronic infection by gram-negative bacteria, or the inability to effectively clear endotoxin, can elicit neurodegeneration [87]. Sustained peripheral immune reactivity to endotoxin can bolster immune cell memory, trafficking, and communication that may contribute to this neuronal vulnerability through an exaggerated inflammatory response. Lipopolysaccharide (LPS) endotoxin is found on the outer membrane of gram-negative bacteria, and the main receptor for LPS, TLR4, is preferentially expressed on microglia [88]. TLR4 activation can elicit NF-kB activation of a broad pro-inflammatory transcriptional response, including pro-inflammatory cytokines. Rats given LPS injections exhibit a pro-inflammatory response, upregulation of APP and β-secretase, downregulation of Aβ clearance, and cognitive impairment, suggesting a role of chronic inflammation in promoting AD pathology [89] (Fig. 2). Studies of AD patients have shown colocalization of endotoxin with Aβ plaques [90], in addition to blood and brain endotoxin levels that are two-to-threefold higher compared to controls [91]. Again, there is evidence of a GxE interaction, where individuals with the *APOE4* allele are more sensitive to environmental insults, in this case LPS endotoxin, in a manner that may further exacerbate their risk for AD [92]. A breadth of research is emerging that supports the role of gut–brain-axis in AD. Early predictive pathology of AD is posited to take place in the gut as a result of microbiota dysbiosis that increases intestinal permeability, activates immune cells, and impairs the blood–brain barrier (BBB) [93]. This is thought to alter cell signaling in the brain, as well as permitting the influx of peripheral immune cells and inflammatory cytokines into the brain [93]. One longitudinal study found that individuals with inflammatory bowel diseases, including Crohn’s disease and ulcerative colitis (UC), have a greater risk of dementia [94]; however, a retrospective cohort study found that only UC was significantly associated with dementia [95]. There is also evidence that AD patients exhibit altered profiles of gut microbiota [96]. Chronic viral infections, including influenza and respiratory tract infections [97], have also been suggested to play a role in the risk of AD onset and progression. Human herpesvirus (HHV) infection is posited to elevate risk and accelerate disease as a consequence of increased Aβ deposition, as seen in a 5XFAD mouse model and 3D human neural cell culture infected with herpes simplex virus type-1 (HSV-1), HHV-6A, and HHV-6B [98]. Furthermore, the chronic pro-inflammatory milieu set by human immunodeficiency virus (HIV) is consistent with the increased risk of neurological symptoms in these patients, such as HIV-associated neurocognitive disorders and HIV-associated dementia [99]. Thus, the “inflammatory-infectious hypothesis” of AD is gaining increasing support over the “amyloid cascade hypothesis” and is beginning to inform the search for therapeutic strategies for AD.

### Therapeutic strategies targeting inflammation in AD

Since the first description of AD over 100 years ago, efforts to identify strategies that would slow, or halt, disease progress have generated limited success. The most common treatment strategies include cholinesterase inhibitors and *N*-methyl d-aspartate (NMDA) antagonists that serve to modulate the cognitive and behavioral symptoms of AD. The search for improved, accessible AD treatment strategies and early disease detection remain driven by the unrelenting nature of AD progression and the associated health and economic burden (USD$355 Billion in 2021) [3]. Thus, earlier detection carries with it the hope for preventing or significantly delaying the onset and progression of debilitating clinical features. The prodromal phase of AD is characterized by mild cognitive impairment, depressive symptoms, and pathophysiological changes to biomarkers in cerebrospinal fluid (i.e., P-tau217, Aβ oligomers) that may precede neuron loss and dementia by as many as 25 years [20, 100]. As such, neural injury and brain changes are likely to occur decades before onset of overt cognitive impairment, at which point, treatment strategies targeting the mechanisms underlying disease risk may prove ineffective in halting disease progression. Therefore, identifying the window of optimal clinical intervention is paramount.

One emerging strategy, directed at early disease stages, seeks to target Aβ aggregation with the prediction that reduced Aβ would retard disease progression and ameliorate symptoms. In 2021, the USFDA accelerated approval of the first disease-modifying immunotherapy for AD, Aducanumab—a human monoclonal antibody that selectively targets Aβ aggregates and reduces soluble and insoluble Aβ in a dose-dependent manner [101]. Approval of Aducanumab is not without controversy, as results from phase III trials (ENGAGE: NCT 02477800; EMERGE: NCT 02484547) lack unequivocal evidence of the drug’s safety and efficacy [102, 103], which, despite these results, is proposed to cost individual patients USD$56,000 annually [103].

Studies of nonsteroidal anti-inflammatory drug (NSAID) use in Tg rodent models of AD (14 studies; reviewed in McGeer and McGeer [104]) have largely found that NSAID use reduces neuroinflammation and microglial activation through inhibition of cyclooxygenase (COX) isoforms and reduces Aβ_42_ accumulation through γ-secretase modulation [104]. Epidemiological studies and meta-analyses have shown that long term use of NSAIDs may be protective against cognitive decline and AD risk; however, these results are not consistently corroborated in the literature, and most clinical trials predicated on encouraging epidemiological and model system data have failed to support a beneficial role for NSAID use in the treatment or slowing of AD [104, 105]. These trials have been relatively small and have included patients already presenting with mild to moderate cognitive decline. Reviews by McGeer and McGeer [104] and Villarejo-Galende et al. [105] have highlighted these inconsistencies. Many reasons could underlie the different results, including study size, population, differences in study design, or missing the window of optimal therapeutic intervention [106]. It is also likely that immune pathology in AD is more complex than COX-2-mediated pathophysiology and NSAIDS are simply not the right agents to target dementia-causing inflammation in the brain.

Interestingly, the NSAID derivative, and γ-secretase modulator, CHF5074 has shown promise in cell and rodent models of AD; CHF5074 treatment suppressed expression of pro-inflammatory markers (TNF, IL-1β, and iNOS), increased expression of anti-inflammatory markers (MRC1/CD206 and TREM2), and demonstrated favorable reductions in plaque and NFT formation, neurodegeneration, neuroinflammation, and cognitive deficit [107–111]. CHF5074 (also known as Itanapraced or CSP-1103) has gone through phase II trials (NCT01303744; NCT01602393; NCT01723670) [112], and preliminary studies of human subjects have shown that CHF5074 treatment is associated with a reduction in neuroinflammation [113]. However, one study found that cognitive improvement was restricted to *APOE4* AD patients [114].

While there has been a shift in focus toward the development of pharmaceuticals that target neuroinflammation in AD [112], emerging data also suggest that non-traditional, anti-inflammatory treatments may display ameliorative properties. For example, curcumin, a polyphenol found in turmeric, exhibits anti-inflammatory, antioxidant, and anti-protein aggregation effects. In murine and cell models, curcumin has been shown to suppress microgliosis, inhibit the production of pro-inflammatory cytokines (IL-1β, IL-6 and TNF), reduce oxidative damage, and reduce Aβ deposition [115, 116]. In small human trials, curcumin has been shown to decrease Aβ_42_:Aβ_40_ ratio and protect against AD and cognitive decline, with no discernible side-effects (34–96 participants; 1 epidemiological study of 1010 participants) [117–120]. Trials employing formulations that increase the bioavailability of curcumin (i.e., Longvida®, Theracurmin®) have seen even greater improvements in mood and cognition, as well as decreases in Aβ levels in sera, as well as Aβ plaque and NFT accumulation in brain regions modulating mood and memory [119, 121–123]. These studies agree with data from cell and rodent models, in which curcumin treatment was consistently associated with a decrease in Aβ levels, inflammation, microglial activation, and cognitive impairment (7 in vitro studies, 7 in vivo studies; reviewed in Mandal et al. [124]). Collectively, these studies underscore the therapeutic potential of curcumin.

The next steps in exploring the value of immunomodulation or anti-inflammatory compounds in preventing or slowing AD onset are not immediately clear. However, the established roles of genetic and environmental factors in modulating inflammatory response and AD risk suggest they may remain a target of therapeutic value. Therefore, substantial follow-up studies may be necessary to re-evaluate the prophylactic and therapeutic efficacy of these strategies in trial participants before the onset of cognitive decline or neurodegeneration. As such, future clinical trials for AD would greatly benefit from the knowledge of the timeframe in which clinical intervention may be most effective.

## The genetic architecture of Parkinson disease

Following AD, Parkinson disease is the next most common age-related neurodegenerative disorder, having affected over 7 million people worldwide in 2020 [125]. As seen in multifactorial diseases, the incidence of PD displays a marked sex bias, with risk 1.5–2 times higher for men [126]. However, despite a similar duration of disease, women appear to experience more rapid progression and a lower survival rate [127]. The pathological hallmarks of PD include loss of midbrain, dopaminergic (mbDA) neurons in the substantia nigra pars compacta (SN) and the appearance of intraneuronal Lewy bodies (LB)/neurites that contain aggregates of misfolded α-synuclein. In patients diagnosed with PD, this manifests as progressive loss of motor control, including resting tremor, bradykinesia, rigidity, and loss of postural reflexes [128]. Although the average age of onset of these symptoms is between 50 and 60 years of age [4], by the time motor symptoms present and permit a clinical diagnosis, patients have already lost 40–60% of mbDA neurons [128]. Consistent with these observations is a swath of prodromal features that have been identified in patients, including sleep and mood disorders, constipation, and progressive anosmia [129]. These prodromal features can precede the onset of motor symptoms by 5–20 years [129].

Akin to AD, PD is often referred to as “familial/monogenic” or “sporadic/idiopathic” in the clinical literature. However, both forms represent multifactorial diseases with risk and progression modulated by both genetic and environmental factors, resulting in clinical phenotypes of variable penetrance. Familial PD accounts for approximately 15% of cases [130]; it is associated with rare variants of high penetrance and autosomal dominant or recessive modes of transmission. In total, ≥ 15 genes have been identified as harboring mutations that result in familial PD or PD-related disorders [130]. Consistent with its role in the formation of LB, the gene encoding α-synuclein (*SNCA/PARK1/PARK4*) has been consistently implicated in PD risk. Variants that promote α-synuclein misfolding [131] and overexpression [132], or events that result in gene amplification [133], have been linked to PD risk. Other risk loci have been identified in familial PD [134, 135]: *PRKN/PARK2* (Parkin; an E3 ubiquitin ligase), *PINK1/PARK6* (PTEN-induced putative kinase 1), and *DJ-1/PARK7* (Protein Deglycase) are involved in mitochondrial and mitophagy pathways, whereas *LRRK2/PARK8* (leucine-rich repeat kinase 2; dardarin) and *GBA* (β-glucocerebrosidase) are involved in lysosomal and membrane trafficking pathways [136].

Sporadic PD is a comparatively late-onset disease, in which risk is influenced by a spectrum of common variants with lower penetrance and the effects of environmental insult. Common genetic variants are thought to explain 16–36% of the heritable risk of PD [4]. To date, GWASs and meta-analyses have implicated nearly 7.8 million SNPs and 90 risk loci, including *SNCA, GBA,* and *LRRK2*, which are also implicated in familial PD [4]. Databases such as Gene4PD (http://genemed.tech/gene4pd) and PDGene (http://www.pdgene.org/) provide a comprehensive repository of risk loci and variants that are associated with PD.

Our ability to detect patterns of similarity in disease are impeded by the combinatorial effects of such a large list of risk loci, and their interactions with substantial environmental/lifestyle differences among human populations. PD pathology is extensively studied, and has given rise to a range of hypotheses involving protein aggregation, mitochondrial dysfunction, oxidative stress, and prion-like transmission [137, 138]. Common to all proposed mechanisms of disease, however, is an undercurrent of inflammatory response—both as a pathological mechanism and risk factor.

### The role of inflammation in PD pathology

**Fig. 3:**
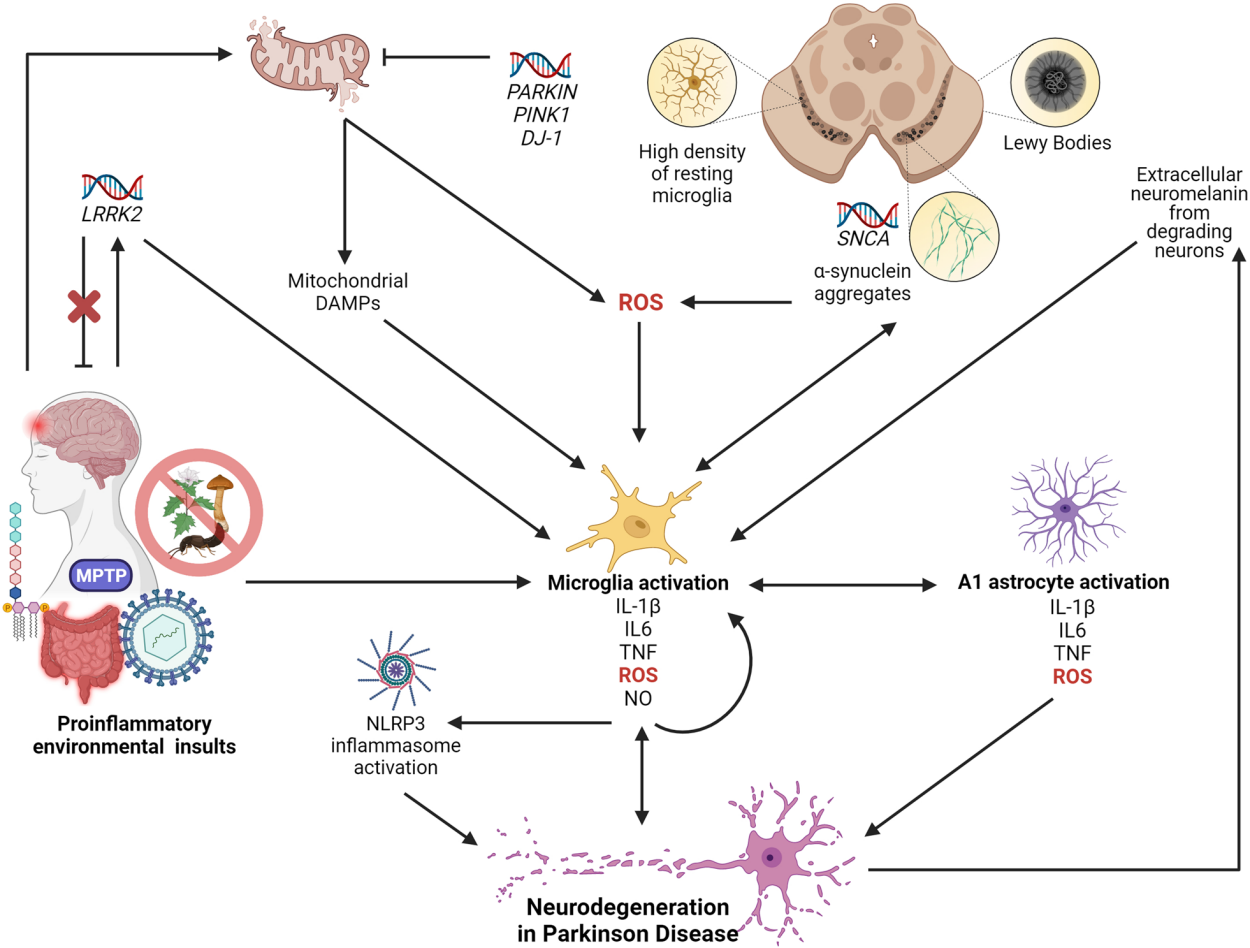
The role of neuroinflammation in Parkinson disease (PD). In PD, the SN is preferentially vulnerable to neuron loss. The high density of resting microglia in this region (12% of cells—the highest in the brain) is thought to render the SN vulnerable to the effects of a robust inflammatory response. Furthermore, neurodegeneration in the SN can cause a feed-forward loop, where, upon neuronal degradation, the high concentration of neuromelanin in the SN is released into the extracellular space and is cleared by activated microglia. Microglia activation, and the subsequent release of pro-inflammatory cytokines, ROS, and NO, is also a central event in the onset of neurodegeneration in PD. Like in AD, pro-inflammatory microglia release cytokines that cause a feed-forward loop of chronic microglia activation, activate the NLRP3 inflammasome, and facilitate cross-talk with A1 astrocytes, all of which coalesce on neurodegenerative pathways. In PD, microglia can be activated upon detection of PAMPs, DAMPs, and other pro-inflammatory molecules as a result of environmental insult—including gut dysbiosis and sustained exposure to LPS endotoxin, viral infection, TBI, MPTP, and pesticides. These pro-inflammatory insults can result in the overexpression of *LRRK2*, which is also pro-inflammatory and can modulate cytokine production. Mutations in *LRRK2* can modulate PD risk, but gene knockouts impair microglia activation and are protective against pro-inflammatory environmental insults. Environmental insults further activate microglia through mitochondrial-mediated toxicity. Pesticides inhibit NADH dehydrogenase, which can lead to the production of ROS and mitochondrial DAMPs, both of which activate microglia. *PARKIN, PINK1*, and *DJ-1* play roles in the clearance of damaged mitochondria, and mutations in these genes can prevent proper clearance and the production of ROS and DAMPs. Pathological hallmarks of PD are aggregates of SNCA surrounded by Lewy Bodies. SNCA mutations, amplification, and overexpression, can result in the formation of these protein aggregates, which are identified and cleared by DAMP-sensing microglia. SNCA can also produce ROS, which interacts with the P2X7 receptor on microglia

Dopaminergic neurons of the SN are preferentially vulnerable in PD; thus, it is worth noting that these neurons are also preferentially vulnerable to the effects of an inflammatory response. Several factors appear to conspire to elevate neuronal vulnerability. Compared to the rest of the CNS, studies of adult mice have shown that the SN has the highest density of resting microglia (which account for 12% of cells in this region) [139] (Fig. 3). As outlined above, stimulation of microglia can release pro- or anti-inflammatory cytokines that subsequently activate other mediators of inflammation, growth, and repair. Like in AD, cellular communication between microglia and astrocytes serves to maintain homeostatic balance in the CNS, and pro-inflammatory cytokine production by activated microglia activates A1 astrocyte signaling cascades (Fig. 1) associated with synaptic destruction and neurotoxicity in PD [39].

Normally, the SN contains a low ratio of astrocytes to microglia, potentially contributing to SN DA neuron vulnerability to ROS, protein aggregation, and other initiators of the inflammatory response [29]. SN vulnerability in PD is further exacerbated by high concentrations of neuromelanin—the pigmented by-product of catecholamine catabolism for which this region of the brain was named. Under normal conditions, neuromelanin plays a neuroprotective role, as the main storage molecule in SN DA neurons for iron and other metals [140]. Detection of extracellular neuromelanin in the degenerating DA neurons of PD patients activates microglia, which triggers pro-inflammatory pathways (i.e., NF-κB and Mitogen-Activated Protein Kinase; MAPK), and results in neuromelanin engulfment [141] (Fig. 3). Evidence suggests that when extracellular neuromelanin is phagocytosed by microglia, iron is released from neuromelanin and produces ROS through Fenton reactions with H_2_O_2_ generated by the deamination and autoxidation of DA [140, 142]. This is thought to further accelerate oxidative stress and neurotoxicity in the PD brain. Studies of PD patient serum and post-mortem human and mouse brain tissues have revealed increased levels of activated microglia and pro-inflammatory cytokines [143, 144], destructive A1 astrocytes [39, 145] (Fig. 3), and infiltration of CD8+ and CD4+ T lymphocytes [146]. Neuroimaging studies have further confirmed the involvement of an inflammatory response in the brain and its contribution to DA neuron loss in PD patients [147]. Therefore, it should be no surprise that genetic risk factors in PD may impact the likelihood and extent of inflammatory response, and that environmental factors that promote/suppress inflammatory response may modulate PD risk/progression (Fig. 3).

### Genetic mediators of inflammatory response in PD

PD risk loci exert their effect through variable mechanisms of action; however, the consequential activation and persistence of an inflammatory response represents the emergence of a common theme. Many PD mutations are now believed to elicit this response through pathways involving oxidative stress [148], and numerous pan-ethnic PD risk loci (i.e.,* DJ-1*, *GBA* and *HLA*) have functional roles in the immune system [149]. Several genes implicated in PD play direct roles in immune/inflammatory response, and common genetic risk variants have been shown to convey risk of both PD and autoimmune diseases [150]. Furthermore, GWASs and meta-analyses suggest a strong involvement of the innate and adaptive immune systems in genetic susceptibility to PD [151, 152]. However, in contrast with AD, many PD risk loci currently lack such a direct attribution.

As part of its role as a major driver of PD pathology, mutant *SNCA* elicits a proinflammatory response. Mutations resulting in SNCA overexpression and aggregation can trigger microgliosis, since SNCA aggregates act as DAMPs that activate pro-inflammatory microglia, and promote the production of pro-inflammatory cytokines, such as IL-1β [153] (Fig. 3). While there is some evidence of T-cell reactivity to nitrated SNCA epitopes (this post-translational modification is not recognized as a self-protein) in early PD [154], studies assaying anti-SNCA B-cell antibody levels in PD patients present discordant data [155]. Aggregation of SNCA is also thought to induce an inflammatory response as a result of ROS production (Fig. 3), through interactions with the microglial P2X7 receptor [156]. As in AD, ROS production and oxidative stress in PD are both inducers and consequences of an inflammatory response, and further contribute to neurotoxicity.

ROS, and subsequent inflammation, can also be a consequence of mitochondrial damage. PD risk loci *PINK1* and *PRKN* are involved in the clearance of dysfunctional mitochondria, but mutations in these genes can impair this function and result in the production of ROS and mitochondrial-DAMPs that can activate an immune response [157] (Fig. 3). This response, characterized by increased levels of IL-6, is thought to be activated in a CGAS (cyclic GMP–AMP synthase)/STING (stimulator of interferon genes)-dependent manner [157]. PRKN and PINK1 also share roles in the modulation of inflammatory cytokines [149], and transcriptomic studies have found that *Pink1*^*−/−*^ mice show altered gene expression profiles of genes involved in innate immunity [158].

The role of an inflammatory response in PD onset can also be influenced by the *LRRK2* risk locus, since high levels of *LRRK2* expression is seen in human peripheral blood mononuclear cells [159] and murine microglia [160]. In addition to roles that overlap inflammatory response, such as mitochondrial function, vesicle trafficking and endocytosis, retromer complex modulation, and autophagy, it is thought that LRRK2 may modulate cytokine production in both a TLR-dependent and TLR-independent manner [161]. As such, mutations at this locus could increase susceptibility to infection and inflammation (Fig. 3). A transcriptomic study of PD patients with *LRRK2* mutations found disruptions in pathways involved in immune response signaling, MAPK signaling, apoptosis, and mitochondrial oxidation [162]. LRRK2 is thought to be pro-inflammatory; in fact, mouse models that are genetically predisposed toward developing PD, but lack *Lrrk2*, show impaired microglial activation and LB formation [163]. *Lrrk2* deficiency has also been shown to be protective against certain environmental insults, such as chemical toxins and viral inducers of inflammation [164, 165] (Fig. 3). Collectively, these studies establish a common theme that genetic predisposition toward inappropriate modulation of an inflammatory response may elevate long-term disease risk.

### Environmental mediators of inflammatory response in PD

Several features of PD highlight the potential for combined genetic and environmental roles in disease, including variable age of onset and variable expressivity of disease phenotypes. Exposure to chemical neurotoxins such as 1-methyl-4-phenyl-1,2,3,6-tetrahydropyridine (MPTP) results in selective destruction of DA neurons in the SN and permanent PD pathology, albeit lacking LB inclusions. MPTP, a by-product in the synthesis of the opioid desmethylprodine (1-methyl-4-phenyl-4-propionoxy-piperidine; MPPP), was first identified as an irreversible inducer of rapid-onset PD pathology in humans who had intravenously injected the chemical as a contaminant of illicit narcotics [166]. Glial cells metabolize MPTP to 1-methyl-4-phenylpyridinium (MPP+), which is taken up by DA neurons. This results in in oxidative stress, mitochondrial damage, and neuron death by inhibiting complex I (NADH-dehydrogenase) of the electron transport chain [167] (Fig. 3). In these cases, patients showed evidence of microglial activation that persisted years after drug clearance, in addition to neuromelanin seen both extracellularly and within microglia (Fig. 3). Consequently, MPTP is used to experimentally induce symptoms of PD in animal models. These models have further illuminated how inflammation may contribute to PD, by identifying that CD4+ T lymphocytes contribute to neurodegeneration through the FasL pathway [146].

Akin to the mechanism of action of MPP+, rotenone induced neuron death is associated with inhibition of NADH-dehydrogenase, and contributes to PD pathology through oxidative stress, ROS production, and inflammatory response (Fig. 3). Rotenone is a pesticide, insecticide and piscicide, exposure to which is also associated with increased risk of developing PD (OR = 2.5) [168]. Exposure to other pesticides, such as Paraquat, also cause oxidative stress and the onset of a pro-inflammatory milieu, and is similarly associated with increased risk of PD (OR = 2.5) [168].

In addition to chemical exposures, there is substantial evidence that the strong inflammatory response associated with TBI is also a risk factor for PD. Years of athletic participation in contact sports are associated with an elevated risk of developing parkinsonism and Lewy Body Disease (OR = 1.30 per year) [169]. As previously discussed, studies highlighting elevated levels of inflammatory biomarkers, specifically IL-6 and TNF, demonstrate that a prolonged inflammatory response follows TBI [72, 83] (Fig. 3). This response is thought to cultivate a pro-inflammatory milieu that renders mbDA neurons vulnerable to neurodegeneration. Indeed, among veterans and military personnel, TBI is associated with a 56% increase in PD risk [170]. Interestingly, mouse models with TBI have been shown to induce HIF-1α-dependent overexpression of the pro-inflammatory PD risk locus *Lrrk2* [171] (Fig. 3). Similar observations have supported an association between inflammatory biomarkers and PTSD in PD [83, 172]. Patients with PTSD are at an elevated risk for PD, alluding to an insidious role of chronic stress in PD risk [173]. Consistent with these data, several studies of humans and rodents have shown that stress increases an individual’s lifetime risk of PD [174–176]—observations that are consistent with the known correlation between cortisol levels and increased PD risk [177].

Inflammation associated with viral and bacterial infections is also an increasingly appreciated risk-factor for PD. Infectious burden, measured by antibody titers to cytomegalovirus, Epstein Barr virus, HSV-1, *Borrelia burgdorferi*, *Chlamydophila pneumoniae*, and *Helicobacter pylori* has been shown to be elevated in PD patients and associated with increased levels of serum SNCA, IL-1β, and IL-6 [178]. In a mouse model infected with H5N1 influenza, microglia activation was noted 90 day post-infection, in addition to SNCA aggregation, phosphorylation at Ser129 (pS129 α‐Syn), and degeneration of DA neurons [179] (Fig. 3). Human macrophages stimulated by LPS and IL-1β upregulate SNCA in a concentration-dependent fashion [180] (Fig. 3). Murine models given LPS injections display increased levels of pro-inflammatory cytokines and microglia activation, loss of DA neurons, and induction of PD phenotypes [181, 182] (Fig. 3). Interestingly, there is evidence to suggest that intranasal LPS can activate microglia and inflammatory cytokines and trigger SNCA overexpression, aggregation, and pS129 α‐Syn in the olfactory bulb—a response that spread to the SN and striatum of mice within 6 weeks, resulting in PD pathology [183]. The “endotoxin hypothesis of neurodegeneration” postulates a dual-hit hypothesis for PD, where elevated endotoxin plus α-synuclein aggregation results in neurodegeneration [87].

Increasing evidence supports the idea that PD pathology can begin in the enteric nervous system (ENS) and spread to the brainstem via the vagus nerve. It is thought that intestinal stimuli modulate signaling pathways in the brain as an effect of vagal afferent signaling (reviewed in Houser and Tansey [184]). This model of pathogenesis posits that intestinal inflammation triggers an initial immune response resulting in gut dysbiosis, increased intestinal permeability, and increased expression and aggregation of α-synuclein. In support of this model, PD patients display LB pathology in intestinal enteric nerves and increased intestinal permeability [185]. Chronic intestinal inflammation and permeability may promote systemic inflammation, which can increase BBB permeability, permit the influx of peripheral immune cells into the brain [184], and result in neuroinflammation/degeneration (Fig. 3). There is also a growing body of evidence demonstrating that risk of PD in patients with chronic gut inflammation, such as in Crohn’s disease and UC, may be 20–90% higher than those without inflammatory bowel disease [186]. These hypotheses are further supported by the incidence of gastrointestinal complications in as many as 80% of PD patients [184].

While more rigorous studies of these mechanisms are required, they are collectively posited to elicit a chronic neuroinflammatory response that significantly contributes to the pathogenesis of progressive neurodegeneration in PD [187]. Ageing is thought to exacerbate these effects since oxidative damage, and the subsequent inflammatory response, is expected to naturally increase over time in these already vulnerable regions of the brain [2]. Proinflammatory processes are inherent in the pathological development of PD and the effects of genetic and environmental risk factors; thus, inflammation itself may serve as a risk factor for the onset and progression of PD.

### Therapeutic strategies targeting inflammation in PD

Presently, there is no cure for PD. In addition to physical and occupational therapy, individuals with PD are most often prescribed a form of levodopa. Levodopa is as DA precursor that is used to supplement DA in PD patients, since it can cross the BBB. While levodopa is considered the gold-standard in PD treatment, its efficacy diminishes over time, and prolonged use with increasing effective dose can result in significant side-effects, including levodopa-induced dyskinesia. Current treatment strategies are only employed upon the clinical presentation of PD, by which point, 40–60% of mbDA neurons have already been lost [128]. Furthermore, the economic impact of clinically defined PD is great, costing Americans nearly USD$52 billion in 2018 alone [188]. As such, the prodromal phase of PD may be the most medically and cost-effective window of therapeutic or prophylactic intervention.

Localized pro-inflammatory response, increased oxidative stress, and selective DA neuronal damage, are all drivers of PD progression. This observation has sparked interest in the potential ameliorative capacity of anti-inflammatory therapies, pharmaceuticals, nutraceuticals, and behaviours. While rigorous follow-up studies are necessary to evaluate the efficacy and mechanism of action of these approaches in preventing or slowing the progression of PD, they contribute to a common narrative that reinforces the potential role of inflammation in PD risk. Consequently, there is an increasing body of literature evaluating the therapeutic value of impeding inflammatory response in mitigating and treating PD. As with AD, epidemiological, model system, and clinical intervention paradigms have been employed with variable results.

Several emerging therapeutic strategies in trials for PD set out to target the endogenous proinflammatory response [189]. Glucagon-like peptide 1 receptor (GLP1R) agonists have recently emerged as valuable neuroprotective agents with potential therapeutic value to PD. Recent work has revealed that NLY01, a potent GLP1R agonist, acts to block A1 astrocyte activation by microglia [190]. In doing so, it reduced DA neuron loss and behavioral deficits in an SNCA–PFF (preformed fibril) PD mouse model, as well as prolonging life and reducing behavioral deficits in the hA53T *SNCA* PD mouse model [190]. In fact, multiple GLP1R agonists are currently being evaluated in clinical trials (Exenatide—Phase 3 NCT04232969; Liraglutide—Phase 2 NCT02953665, Lixisenatide—Phase 2 NCT03439943; NLY01—Phase 2 NCT04154072) [189].

While some epidemiological studies and meta-analyses have found an association between regular NSAID use (particularly ibuprofen) and reduction in PD risk [191–193], most studies report non-significant therapeutic effects alongside considerable risk of heart attack, stroke, gastrointestinal bleeding, and kidney problems [194]. One recent study of *LRRK2* PD mutant and PD-risk variant carriers demonstrated that regular NSAID use was associated with reduced PD risk [195]. However, additional clinical trials are warranted in evaluating the potential of alternative immunomodulatory therapeutic approaches in PD. Here again, identifying the optimal timespan to therapeutically target neuroinflammation in these disorders may be paramount for the success of future clinical studies.

Curcumin also represents an intriguing prospect for PD risk reduction. Curcumin displays neuroprotective effects in animal and cell models of PD [124, 196–198] and has been shown to improve motor symptoms in a mouse model of PD overexpressing *Snca* [199]. Curcumin is most commonly used for culinary and medicinal purposes in Southeast Asia, India and China, where, interestingly, PD risk is significantly lower than in North America, Europe, Australia, and South America [200]. While this evidence is circumstantial, it suggests value in both epidemiological studies and clinical trials to evaluate the therapeutic potential of curcumin for PD risk reduction.

However, both epidemiological and animal studies have implicated caffeine intake as a risk reducer for PD (reviewed in Ren and Chen [201]): in an MPTP mouse model, intraperitoneal administration of caffeine attenuated microglia reactivity, as well as demonstrating a decrease in loss of DA neurons. Its neuroprotective properties are thought to act through the adenosine 2A receptor, since genetic deletion and pharmacological blockade of this receptor is protective against DA neuron degeneration and rescues synaptic and cognitive deficits in animal models of PD [201]. Furthermore, supporting the increasingly acknowledged role of the gut–brain axis, caffeine is reported to modulate gut microbiota in preclinical PD models [201].

Both genetic and environmental factors clearly modulate neuroinflammation and PD risk; thus, suggesting that targeting the inflammatory response is of prophylactic and therapeutic value. Consequently, additional studies are necessary to identify the critical time window in which this inflammatory response may contribute to disease risk, such that the development and evaluation of interventional strategies may be more robustly undertaken.

## Conclusions and future directions

Here, we have highlighted the roles played by inflammatory dysregulation in sensitizing the risk, onset, and pathology of Alzheimer disease and Parkinson disease—two of the most prevalent neurodegenerative disorders. An inflammatory response to environmental risk factors may be, directly or indirectly, modulated by genetic variation, and may represent a significant component or driver of neurodegenerative disease. Indeed, inflammation appears to provide a potential unifying nexus of neurological disease risk. With this in mind, it appears particularly prudent to monitor the relationship between wide-spread inflammatory response arising in response to the novel coronavirus, SARS-CoV-2, and the incidence of neurodegenerative diseases in the coming decades.

The collective investment in illuminating the environmental and genetic risk factors, pathways, and cell populations involved in neurological disease has generated independently significant biological insights. Arising from these studies, inflammation provides a unifying theme that can be exploited in the pursuit of biologically directed, therapeutic approaches. The extent to which these observations collectively inform emerging strategies for therapeutic intervention for these, or other common disorders, will comprise some of the most anticipated data in the study of common genetic disease over the next decade.

COVID-19 as an emerging inflammatory challenge The COVID-19 pandemic has placed an incredible burden on individuals, societies, and healthcare systems around the world. While immediate concerns are rightfully placed on slowing the spread and evolution of the virus, there are emerging concerns that patients may be at heightened risk for developing a neurodegenerative disorder after fighting the primary COVID-19 infection [3, 202]. There is evidence that a subset of COVID-19 patients experience long-lasting neurological symptoms (i.e., headache, confusion, anxiety, depression, sleep disturbances, and anosmia [203, 204]) that overlap those implicated in the prodromal phase of many neurodegenerative disorders [129, 205]. Moreover, symptoms of neurological conditions have been observed following a COVID-19 infection and comprise post-acute COVID syndrome, including encephalitis, myalgic encephalomyelitis, encephalopathy, acute disseminated myelitis, and ischemic stroke [204]. In fact, post-mortem and animal studies show that COVID-19 can infect neurons and elicit neuroinflammation [206, 207]. Furthermore, patients who suffer from long-haul COVID [204], may experience this chronic inflammation in an exaggerated fashion. Akin to the mechanisms by which other chronic, viral infections have been seen to elicit neurodegeneration, a COVID-19 infection may pose a secondary threat, driving inflammation throughout the lifespan. In line with our fundamental thesis, COVID-19 may represent an emerging environmental risk factor that could trigger inflammatory dysregulation in the CNS and tip the balance toward instigating neurodegenerative pathology, especially in those individuals with underlying genetic predisposition. Although speculative, the possible long-term impact of COVID-19 on neurological disease risk, will necessitate study in the decades to come.

## Data Availability

Not applicable.

## Abbreviations

ABCA1: ATP-binding cassette A1
ABCA7: ATP Binding Cassette Subfamily A Member 7
AD: Alzheimer disease
APOE: Apolipoprotein E
APP: Amyloid precursor protein
Aβ: Amyloid-beta
BBB: Blood–brain barrier
CD206: Mannose Receptor C-Type 1
CD2AP: CD2-Associated Protein
CD33: Sialic Acid-Binding Ig-Like Lectin 3
CGAS: Cyclic GMP–AMP synthase
CLU: Clusterin
CNS: Central nervous system
COX: Cyclooxygenase
CR1: Complement receptor 1
DAMPs: Damage-associated molecular patterns
DJ-1: Protein Deglycase
EAOD: Early onset Alzheimer disease
ENS: Enteric nervous system
GABA: γ-Aminobutyric acid
GBA: β-Glucocerebrosidase
GLP1R: Glucagon-like peptide 1 receptor
GWAS: Genome-wide association studies
GxE: Gene-by-environment
HHV: Human herpesvirus
HIV: Human immunodeficiency virus
HSV: Herpes simplex virus
IL-1β: Interleukin 1β
IL-6: Interleukin 6
IPMK: Inositol Polyphosphate Multikinase
LB: Lewy bodies
LOAD: Late-onset Alzheimer disease
LPS: Lipopolysaccharide
LRRK2: Leucine-rich repeat kinase 2
MAPK: Mitogen-Activated Protein Kinase
mbDA: Midbrain dopaminergic
MPP+: 1-Methyl-4-phenylpyridinium
MPPP: 1-Methyl-4-phenyl-4-propionoxy-piperidine
MPTP: 1-Methyl-4-phenyl-1,2,3,6-tetrahydropyridine
NFTs: Neurofibrillary tangles
NK: Natural killer
NLRP3: NLR family pyrin domain containing 3
NMDA: *N*-Methyl d-aspartate
NO: Nitric oxide
NSAID: Nonsteroidal anti-inflammatory drug
PAMPs: Pathogen-associated molecular patterns
PD: Parkinson disease
PFF: Preformed fibril
PINK1: PTEN-induced putative kinase 1
PRKN: Parkin
PSEN1: Presenilin 1
PSEN2: Presenilin 2
PTSD: Post-traumatic stress disorder
ROS: Reactive oxygen species
SN: Substantia nigra
SNCA: α-Synuclein
SNP: Single-nucleotide polymorphism
STING: Stimulator of interferon genes
TBI: Traumatic brain injury
Tg: Transgenic
TLRs: Toll-like receptors
TNF: Tumor necrosis factor
TREM2: Triggering Receptor Expressed on Myeloid Cells 2
UC: Ulcerative colitis
